# Injury in kite buggying: the role of the ‘out-of-buggy experience’

**DOI:** 10.1186/s13018-018-0818-x

**Published:** 2018-05-02

**Authors:** F. Feletti, E. Brymer

**Affiliations:** 1 Department of Diagnostic Imaging, Ausl della Romagna, S. Maria delle Croci Hospital, Ravenna, Italy; 2 Department of Electronics, Information and Bioengineering, Politecnico di Milano, Milan, Italy; 3 School of Sport, Leeds Becket University, Leeds, UK

**Keywords:** Extreme sports, Injury, Equipment

## Abstract

**Background:**

The purpose of this descriptive, epidemiological study is to classify injury patterns and determine dynamics of injuries, possible causes and preventive measures.

**Methods:**

A questionnaire was filled in by 127 kite buggying enthusiasts in 17 countries. Injuries were classified by type and anatomical site. Incident causes were analysed using the Haddon matrix.

**Results:**

Injuries classified as moderate or severe (AIS score ≥ 2) were sustained by 26% of kite buggy enthusiasts. The most common incident dynamic (61.8%) was the OBE (an acronym for ‘out-of-buggy experience’). Causal factors were largely equipment-related (42.3%), with remaining incidents being equally attributable to environmental and human factors. While upper and lower limbs were equally involved in incidents, the most frequently affected anatomical site was the shoulder (23%).

**Conclusion:**

Kite buggying can be considered a sport with the potential for serious injury. Injury prevention in this sport needs to be approached from several angles and should include the development and adoption of automatic release systems and shoulder guards, the establishment of formal training programs covering the subject of meteorology and the establishment of secure, designated kite buggying areas. Findings from this study are important for two reasons. First, they demonstrate the significance of understanding specific sports when considering health and safety, and second, the study provides specific data for the fast growing extreme sport of kite buggying.

## Background

The use of kites as traction for land carts and other vehicles can be traced to Asia as early as the thirteenth century [22]. In 1827, ‘Charvolant’, a forerunner of contemporary kite buggy, was developed by Pocock, a British inventor, who successfully ‘sailed’ from Bristol to Marlborough. He claimed to have exceeded a speed of 30 kph [30, 32]. Kite buggying as a modern extreme sport, however, was pioneered in the early 1990s by Peter Lynn at Argyle Park in Ashburton, New Zealand. Kite buggying has become ever more popular, and according to the International Federation of Kitesports Organizations (http://ifkitesports.org/), in 1999, kite buggy sales had topped 14,000, and by 2006, more than 140,000 kite buggies had been sold worldwide. In 2006, there were an estimated 200,000 kite buggy pilots, and the discipline is still growing. Kite buggying is recognised as a competitive sport by the International Sand and Landyachting Federation (FISLY) [16].

Despite the continuing popularity and exponential growth of kite buggying, there is limited understanding of injury patterns and rates. An understanding of incident dynamics and injury types and anatomical sites is of fundamental importance to both epidemiology and sports medicine as it allows for the identification of protection and prevention systems and ensures that future research into prevention is guided appropriately. Furthermore, research has shown that sport-specific and context-specific studies demonstrate that attempting to transfer knowledge from one sport to another is problematic and often inaccurate. This paper presents injury patterns in kite buggying and aims to classify participant-reported injuries by type and anatomical site, to describe the possible causes and the dynamics of the incidents and illustrate important factors for the development of preventive measures.

### The sport

**Fig. 1:**
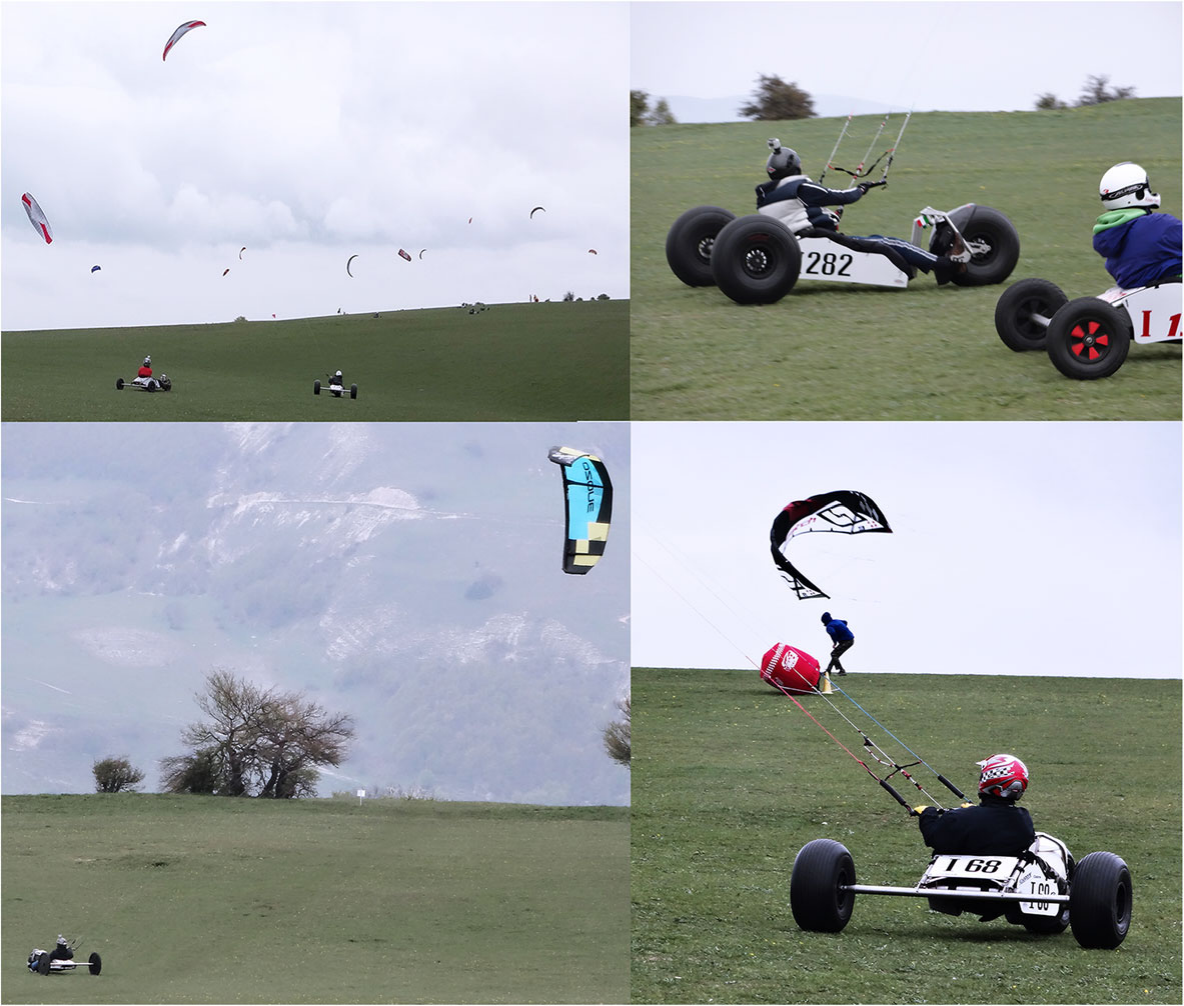
Kite buggying in Monte Petrano, Italian Championship 2013

**Fig. 2:**
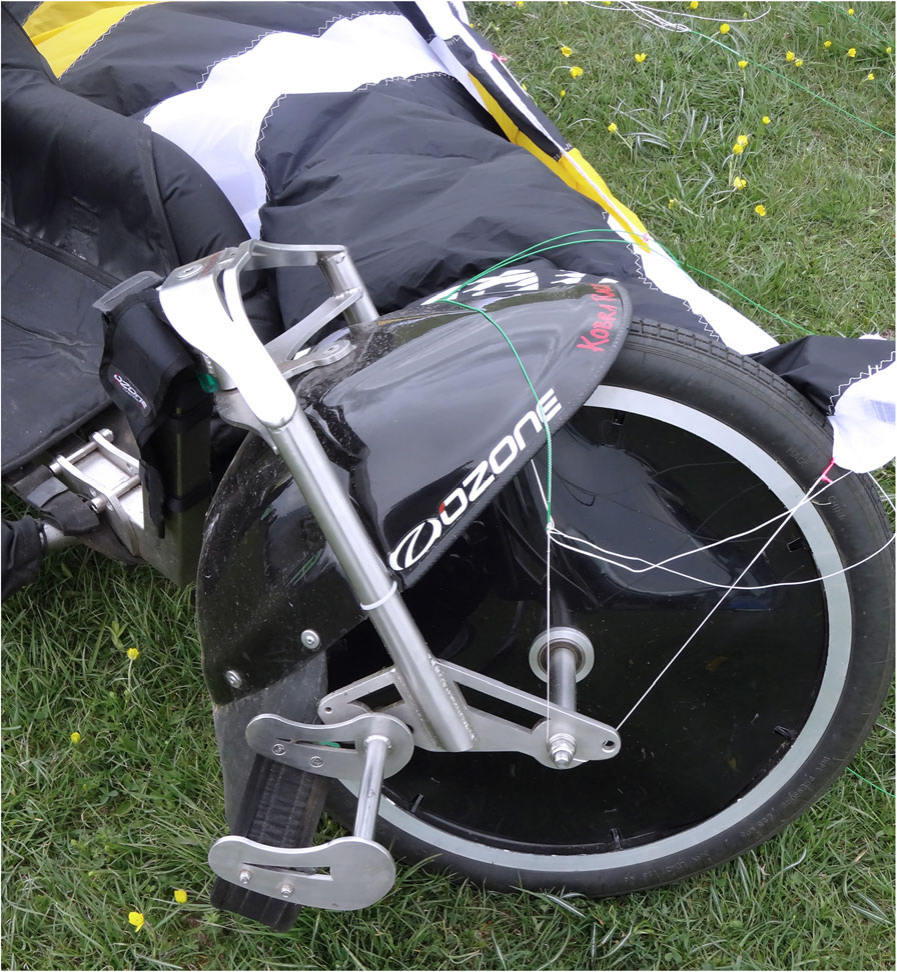
Straps attached to the front wheel allow the kiter to steer the buggy

Kite buggying is considered an extreme sport, where extreme sports are those activities where a mismanaged execution or mistake has the potential to result in death [1]. Infrequent kite buggy incidents have resulted in the death of the participant. For example, as recently as 2009, an experienced kiter died when he and his craft were lifted by strong winds [33]. Kite buggying consists of piloting a buggy (a three-wheeled cart) that is pulled along by a traction kite (Fig. 1). Kites that use foil technology (a cell structure inflated by the wind) are generally preferred over the leading edge inflatables (single skin kite with inflatable bladders providing structure) used in kitesurfing. This is because the sport is practiced on land and does not require a floating wing and because foil kites are cheaper, can be set up faster and are easier to handle. The kite is attached to four lines, 25–30 m long, each with a load capacity of 200 kg. The ‘kiter’ hand-pilots their kite by way of special handles or a control bar; these allow the pilot to adjust the tension on the two lines attached to the trailing edge of the wing and to steer it. The kiter is attached to the kite by means of a harness that spreads the wind force over the hips and lower back. Once sat on the buggy, the kiter inserts their feet into straps that allow them to also steer by way of the front wheel (Fig. 2). Buggies are not equipped with brakes, and the kiter accelerates or slows down by coordinating kite flying manoeuvres with actions on the buggy steering mechanism. Speeds can exceed 100 km/h, and kiters can perform various freestyle aerial manoeuvres such as jumps, 360° spins, reverse landings, sidewinders, pendulum swings and so on. Many countries hold organised rallies, freestyle competitions and course racing events.

### Injury patterns in similar activities

While we have noted that it is important to have detailed information about specific sporting activities as there are inevitably unique characteristics that are not reflected in similar sports, a comparison with similar sports will help determine how these unique characteristics might be understood and managed. Probably the nearest similar sports to kitebuggying are other kitesports, namely kitesurfing and snowkiting. Kitesurfing is a surface water sport involving the use of specific boards, and snowkiting is practiced on snow, using normal freestyle skis or snowboards. Typical injury patterns in kitesurfing happen in the ankle/foot region (28.2%) [24].

On the other hand, the back and knee are very prone to injuries in snowkiting (30.3 and 24.2% of the total number of injuries) where specially designed boots stabilise and protect ankles and feet [10]. However, kitebuggying is a land-based activity and neither kitesurfing nor snowkiting involve strapping the pilot into a large and heavy buggy.

## Methods

The study surveyed international kite buggy sailors over a 21-month period (April 2013–December 2014).

Participants consisted of 127 kite buggying enthusiasts who had been practicing for at least 6 months. Athletes competing in the Italian Kite Buggying Championship (CIKB), held on 27–28 April 2013 at Monte Petrano (PU), Italy, were invited to take part in the study. Participants were also enrolled from the membership of key national federations (see Table 1) and via two kiter web portals: Alienbuggy (www.Alienbuggy.com) and Extreme Kites (http://www.extremekites.com.au).

**Table 1:** Demographic data and information on sporting practices

Gender	M 117, F 8, unspecified 2
Age	Average 39.6, min 19, max 60, SD 10.1
Country	Germany 38, Italy 23, Australia 13, France 17, Spain 1, USA 7, UK 14, Belgium 2, Denmark 2, Netherlands 3, Brazil 1, Costa Rica 1, Ireland 1, Japan 1, Mexico 1, Uruguay 1, New Zealand 1
Affiliation	CIKB 20, GPA 26, FFCV 12, AEKB5, SPKA 3, BCH 3, other (21 associations) 19, none 39
Experience (years)	> 7, 52; 3–7, 55; < 3, 15; unspecified, 5
Rating	Instructor 11, advanced 61, intermediate 48, beginner 5, unspecified 2
Kite type usually used	Foil 118, leading edge inflatable 6, with battens 1, unspecified 2
Days of practice/year	Min 1.5, max 216, average 42.8, SD 39.9, mean 38.5
Session duration (hours)	Average 4.2, SD 2.2, min 1, max 3, mean 4

*AEKB* Asociación Española de Kite Buggy, *BCH* Buggy Club Holland, *CIKB* Campionato Italiano Kite Buggying, *FFCV* Fédération Francaise de Char à Voile, *GPA* German Parakart Association

Kitesailing E.V., SPKA Scottish Power Kite Association

Participants were asked to respond to a questionnaire designed to collect demographic data and data about injuries, execution and near misses. The questionnaire was divided into three separate parts: the first section required basic demographic information (age, sex, country, any sports club membership), years of experience and self-evaluation of personal skill level based on the categories beginner, intermediate, advanced and instructor levels used in other kitesurf and snowkite studies [20, 24]. Information about the kite buggy model and type typically used was also collected. The second section asked participants whether they had been injured while kite buggying. An affirmative response triggered further questions in order to identify the anatomical site and injury type. To encourage full disclosure, quantitative and qualitative elements were combined that asked participants to both respond to a predetermined list of possibilities and encouraged full and detailed description of the type and seriousness of the injury. Participants were also asked to specify any medical treatment, whether and for how long they were forced to abandon the sport and to state any long-term consequences of the incident.

The third section concerned the dynamic of the event: participants were asked to state when it had happened, how strong the wind had been at the time and to give a detailed account of the dynamics and causes of the incident. Respondents were also asked to indicate any damage to the equipment and any collateral damage to property or other persons. They were also asked which causal factors they thought needed attention in order to prevent future kite buggying injuries. Participants involved in more than one incident were asked to respond to the questionnaire for each event, using the automatically created code developed the first time the questionnaire was completed in order to match demographic information. Questionnaires were made available in both paper and electronic form, published on the ExtremeSportMED (www.extremesportmed.org) website in English, German, French and Italian. Data was transferred manually onto datasheets and analysed using descriptive statistics with Wizard Pro 1.3.27 software.

Injuries were classified by anatomic site and type. Only those injuries rated as moderate to severe according to the Abbreviated Injury Scale (AIS score ≥ 2) [2, 3] were taken into consideration because minor injuries such as sprains, lacerations, abrasions and contusions are common in extreme sports and ‘not significant enough to recall’ [19]. The causes of the incidents that had instigated significant injuries were analysed using the Haddon matrix [12], a model broad in scope that is adaptable to extreme sports [34].

## Results

A total of 136 completed questionnaires (124 online, 12 paper) were collected; three online questionnaires were omitted, two because they had been sent without being completed and one because it had been sent twice. Consequently, a total of 133 questionnaires filled out by 127 participants (answer rate 31.7%) were analysed; the general demographic data and sport-related information from section one is shown in Table 1.

Eighty-eight of the participants (69.3%) reported at least one incident, two participants reported two incidents and one participant reported five incidents. However, one case was a near miss that resulted in no injury. A total of 93 injury events were reported.

At least one injury classified as moderate or severe (AIS score ≥ 2) was sustained by 26% (*n* = 33) of the kite buggy practitioners being studied. A total of 39 injuries were sustained in 34 separate incidents (Table 2). Kiters who sustained injuries were not beginners or untrained since 87.8% had been practicing the sport for more than three years; 69.7% of those who sustained injuries rated themselves as advanced or instructor, and 84.8% stated they practiced the sport more than 21 days a year.

**Table 2:** Number of the 39 injuries reported that were classified as moderate to severe in 34 incidents

AIS score (tot. n.)	Anatomic region (tot. n.)	Description of injuries		Number of injuries
3 (9)	Vertebral (7)	Fractures	Cervical (C4-C5)	2
			Dorsal (D9-D12)	3
			Coccyx	1
		Dislocation	Lumbar	1
	Knee (3)	Fracture-dislocation + torn ligaments		2
2 (30)		Meniscus tear		1
	Shoulder (9)	Dislocation		4
		Fracture		3
		Tendon injury		2
	Thorax (7)	Multiple rib fractures		7
	Leg (3)	Fracture		3
	Ankle (4)	Fracture		3
		Ligament rupture		1
	Head (2)	Concussion		2
	Thigh (2)	Quadriceps major muscle laceration		2
	Upper limb (2)	Multiple fractures		1
		Scaphoid fracture		1

*AIS* Abbreviated Injury Scale

## Incident dynamics

Incident dynamics are described in Table 3. In most cases (61.8%), the kite had lifted the athlete out of the buggy, a dynamic that kiters commonly refer to as an ‘out-of-buggy experience’ (OBE).

**Table 3:** Classification of injury events based on incident dynamics and relative causal factors (crash phase) according to the Haddon matrix

Events (*n*)	Dynamics	Description of factors		
		Environmental (*n*)	Human (*n*)	Equipment (*n*)
21	OBE	Hole in the ground (1) Seaweed bank (1) Sudden change in wind direction or intensity (6)	Speed too high (1) Pilot error in steering the kite (2) Error during a change of direction (1)	Kite becomes uncontrollable (8) Line breakage (1)
5	Lifted by kite while not on the buggy	Sudden change in wind direction or intensity (2)	Inattentiveness when launching the kite (1)	Kite becomes uncontrollable (2)
3	Flipped buggy	Large hole in the ground (1)	Kite steering error (1) Error in performing a freestyle manoeuvre(360° spins) (1)	–
2	Collision with equipment	–	Gust of wind (1)	Detached wheel (1)
1	Collision with obstacle	–	The pilot did not see an obstacle (1)	–
1	Involuntary manoeuvre	Sudden gust of wind (1)	–	–
1	Collision between two buggies	–	Poor visibility (1) (during a night-time competition)	–

## Sequential analysis of injury events

### Crash factors

Table 3 classifies injury events on the basis of relative causal factors according to the Haddon matrix. All 34 incidents occurred using foil-type kites, and 59.8% of the incidents occurred with an average wind speed of over 20 kn.

### Pre-crash factors

Pre-crash factors were reported in 13 cases. Most of them (46%) were equipment factors, including the following: too large a kite for the wind intensity (*n* = 3), ill-fitting buggy (*n* = 2) and ill-fitting harness (*n* = 1).

Pre-crash human variables were reported in four cases (30%), namely, speed too high (*n* = 2), overpowered flying style (*n* = 1) and practicing too far from the base (1). Environmental factors were reported in three cases (23%): two were attributable to rough terrain, while one case involved a fast-moving storm front generating strong gusts of wind.

### Post-crash factors

Post-crash factors were reported in five cases. Equipment was the major factor reported (80%) as kiters were unable to disengage from the kite (the consequence being that kiters have, in some cases, been dragged several tens of metres after the initial incident). One case involved an environmental factor in the form of an obstacle (fence) too close to the path that was hit by the athlete on landing after an OBE.

Considered collectively, equipment-related factors were prevalent (42.3%, *n* = 22) while the remaining factors were accounted for by environmental and human factors on an equal basis.

### Losses

#### Human and socio-economic

In 76.5% (*n* = 26) of cases, the reported injuries resulted in an absence from the sport of more than a month; in 14.7% (*n* = 5) of cases, it was less than one month; and in three cases, the time was not specified. Long-term consequences were reported in 20.6% (*n* = 6) of cases. More specifically, these were (1) a case of continued low tolerance to any load on the knee; (2) a case of limited shoulder functionality; (3) two cases of deformity to the shoulder, one being associated with limited functionality; (4) a case of persistent limited movement of the wrist joint; and (5) a permanent disability occurred due to fracture of the last dorsal vertebrae (the kiter stated that he still practices kite buggying). One kiter described experiencing prolonged oculomotor muscle difficulties after a cranial-cervical trauma, a condition resolved through rehabilitation after about a year. In 11.4% of cases, surgery was required.

#### Vehicles and equipment

Two incidents involved buggy damage, one of which, caused by a collision between two buggies, resulted in damage to the buggy receiving the collision and minor injuries to its user. No collateral damage was reported in any of the other cases.

### Prevention targets

#### Equipment

**Fig. 3:**
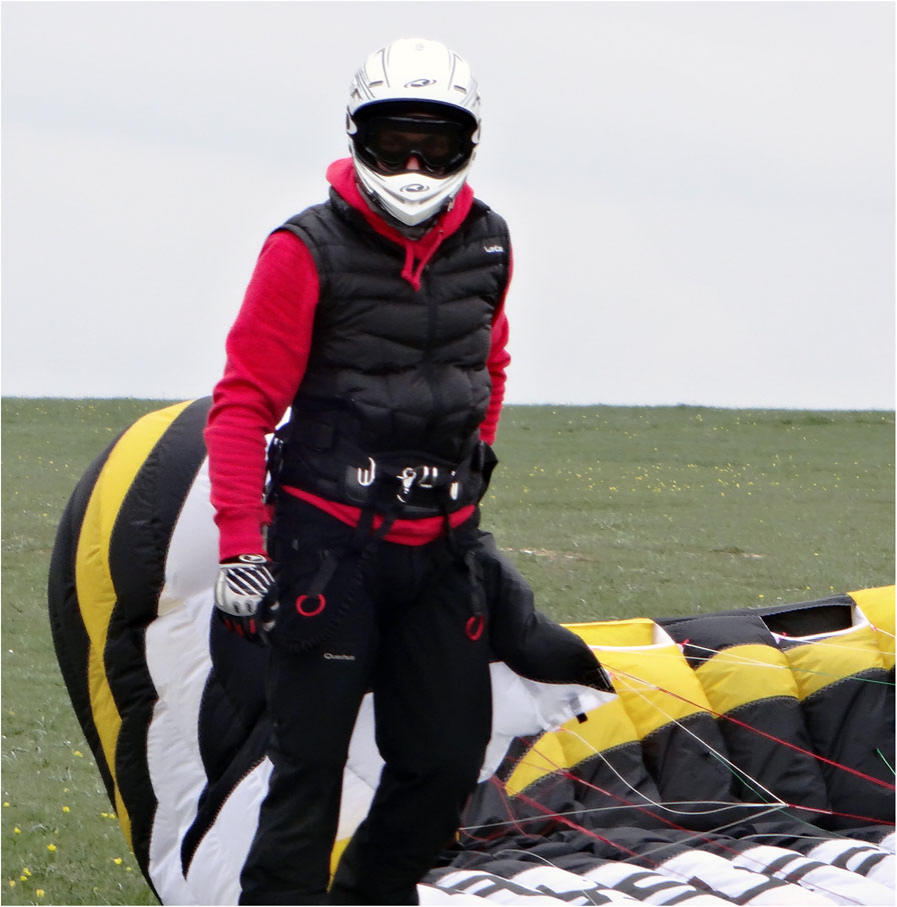
Protective equipment including helmet, protective jacket and wrist protections are the most important injury prevention solutions according to the majority of respondents

With regard to possible injury prevention solutions, the majority of respondents (55.4%, *n* = 56) thought that prevention measures should focus on equipment (see Table 4). Of these, 62.5% (*n* = 35) suggested specific body protection systems and in particular kiters suggested helmet use (*n* = 17) (Fig. 3). The use of more effective quick release systems was recommended by 21.4% (*n* = 12), while 5.3% (*n* = 3) advised utilisation of kites with reduced lift in disciplines such as course racing and amateur activities where no jumps are involved.

**Table 4:** Respondents’ assessment of recommendations to guide future health and safety options (*n* = 101)

Target	Kind	Specific injury prevention solution	Number	
Equipment	Protective	Helmet	17	56
		Protective jacket	3	
		Wrist protections	1	
		Spine protection	5	
		Pelvis protection	2	
		Knee pads	1	
		Leg protections	1	
		Boots	1	
		Not specified	4	
	For sport	Quick release system	12	
		Leash for the kite	1	
		Kites (more stable)	3	
		More fitting kite seats	2	
		Seat belts	1	
		Ergonomic buggies (without sharp edges)	1	
	For training	Development of an interactive video-game-specific training software	1	
Human factor	Training/education	Schools/teaching programs	12	41
		Formal training/practice	13	
		Licencing	3	
		Awareness and ability to judge environmental conditions (wind in particular)	9	
		Knowledge of the location	1	
		Knowledge of rules on priority	1	
		Habit not to use oversized kites	2	
Environment	Organised flying sites		4

#### Human factor

Out of all the participants, 27.7% (*n* = 28) pointed out that the establishment of schools or training programs for beginners would be useful.

#### Environment

Preventive action relating to environmental factors were proposed by 3.9% (*n* = 4) of participants, including the establishment of specifically designated kite buggying sites.

## Discussion

Kite buggying is generally categorised as an ‘extreme sport’, a large and heterogeneous collection of sports which has, overall, enjoyed growing popularity over the last 40 years [4, 14, 25]. Although the exact number of participants is unknown, according to the German Parakart Association Kitesailing, it is a fast developing and international sport (http://www.gpa.de/kbsport). Controlled studies of kite buggying-related injuries have yet to be conducted. Research on incident and injury dynamics is of fundamental importance as it allows for the identification and implementation of sport-specific protection and prevention systems and guides future research. This paper presents kite buggying from a medical viewpoint and classifies participant-reported injuries by type and anatomical site, in order to describe the possible causes and dynamics of incidents and determine the factors underlying preventive measures. The following sections are divided into two parts, injuries and incident dynamics and preventive measures.

### Injuries

Severe injuries (AIS score ≥ 3) accounted for 6.4% of all those reported in the questionnaires. This means that 4.7% of all participants reported severe injuries: a value some 10 times lower than that indicated in a similarly conducted study of BASE jumping injuries which is considered to be the most dangerous extreme sport [19]. Results from this study show that upper limbs and lower limbs were affected similarly; however, the shoulder was, in absolute terms, the anatomical site most frequently affected by injury (23%; *n* = 9). In the kitesurfing study carried out by Nickel et al. [24], shoulder injuries were rare (0.8%; *n* = 1) while ankle/foot injuries were the most frequent (28.2%; *n* = 35). Data provided by respondents in this study showed the ankle to be involved in just 10.2% of all injuries, probably because in kite buggying the kiter is in a sitting position when sailing the buggy which reduces ankle exposure. Traumas were also reported as having taken place while the athlete was standing outside the buggy, especially during takeoff and landing of the kite.

Additionally, some participants described ankle injury dynamics that were kite buggying-specific, such as incidental impact against terrain with the buggy moving.

A study by Moroder et al. [20] found that the shoulder was the most common injury site (21.4%) in snowkiting. Since both kite buggying and snowkiting are land sports, it is likely that falls on hard ground following loss of kite control cause shoulder injuries with similar mechanisms in both disciplines. As the arm movements needed to fly the kite are the same in both disciplines, similar shoulder protection systems could be designed for both sports.

This study also revealed that kite buggying incidents result in a number of rib fractures. As in kitesurfing [18], some arose from the forces transmitted from harness to chest. However, in most cases, rib fractures were caused by falling from a height or being dragged along the ground after an OBE. As observed in studies on kitesurfing [24, 29] and snowkiting [20], kite buggying incidents of this nature can result in serious injuries with lasting consequences. For example, the 2009 fatal kite buggying incident [33] noted above resulted from a kiter and his buggy being lifted by a strong, sudden gust of wind. The kiter was attached to the buggy with a special belt (sometimes used by the most experienced freestyle kiters to ensure the buggy remains attached to them during aerial manoeuvres) and fell from a height of about 10–15 m. Despite the helmet, the rider went into a coma and his head injuries proved fatal.

### Incident dynamics and preventive measures

In this study, the most common dynamic responsible for injury-causing incidents was the OBE.

An ‘OBE’ involves the athlete being lifted from the buggy seat by the force of a kite over which control is impossible, resulting in loss of contact with the vehicle and, therefore, any control over it. OBEs may be caused by sudden gusts of wind, by incorrect coordination of kite flying and buggy driving manoeuvres or by abrupt changes in the speed or direction of the buggy (e.g. due to contact with holes or sandbars). A line break can also make the kite ungovernable and cause an OBE.

In an OBE, and other occasions when control of the kite is lost, the pilot is lifted into the air and is usually injured in the uncontrolled fall back to the ground. Loss of wing control means the pilot is unable to either prepare for landing by using the kite to slow the fall or assume a position that might cushion it. The first body part to hit the ground is usually the one that is injured, and in kite buggying, this is frequently the shoulder.

Equipment emerges as the prime causal factor of incidents. Given the frequency with which the OBE is reported as the cause of serious injury, as in other kitesports [20, 27], the adoption of appropriate ‘smart’ quick release systems might reduce the number and severity of incidents. The current quick release systems require that kiters, when faced with danger, actively disengage.

However, this potentially runs against their natural inclination to remain attached to the vehicle and regain control. Moreover, dangerous situations may be hard to recognise in the early stages (because of the numerous variables involved), and releasing the kite in such situations can be difficult as the chain of events can build up in fractions of a second. For these reasons, ‘smart’ systems, capable of recognising specific hallmarks, such as an increase in load on the kite coupling, may be useful.

One respondent suggested adopting the splitter system. This is a special length-adjustable strap, connected at one end to the quick release activation system worn by the kiter and at the other end to the buggy. In the event of an OBE, the strap becomes taut and automatically activates the quick release system. While this may be a useful OBE safety solution, few kiters are aware of it; indeed, it is estimated that, until 2013, no more than 100 kiters worldwide employed it (http://popeyethewelder.com/).

Results from this study indicated the shoulder as the most frequently injured anatomical site. However, not one of the athletes suggested the adoption of shoulder pads/guards, probably as they were deemed too bulky and movement-restricting. Indeed, any body protection system must be designed to provide the freedom of movement needed to control the buggy; for example, the helmets used in kitesports must provide good peripheral vision, especially when looking up, and should not impede hearing [6, 9]. The introduction of total-depower kites in kitesurfing in 2005 reduced the chances of serious incidents from an out-of-control kite [9, 26]. While the foil kite used in kite buggying is different, this study suggests that the extension of suitable constructive measures, designed to improve safety and stability in foil kites, should be considered. The adoption of ergonomic criteria in constructing sports equipment [28] may also have a role to play: building buggies without any sharp edges and which make it difficult for the athlete to be thrown out may be useful.

Kite and kiter must always be linked by a leash that aids more rapid loss of kite power if the quick release system is activated as this can prevent potential injury to third parties [9]. Lastly, while no injuries have been reported with this mechanism, to avoid any ‘catch in line’ scenario, the adoption of a line cutter ‘hook’ specifically designed for kitesurfing and stowed in a special harness pocket [36] should be taken into consideration.

In addition to equipment, environmental variables also constitute a major risk factor and have been reported as being on a par with human factors. This highlights the importance of specific training programs that cover meteorology and the procedures/conduct to be adopted in different situations. Finally, similar to what has happened in kitesurfing, it may be useful, especially in places where kite buggying is practiced regularly, to set up obstruction-free designated areas where access is denied to those not actually engaged in the sport.

## Limitations

The answer rate for this study was influenced by participant concerns that focusing attention on injuries might undermine the image of kite buggying and lead to legal restrictions on the sport. Nevertheless, a good sample was obtained by approaching the sport from within [8]; the fact that the promoter of the study was himself a kiter made it possible to present the questionnaire directly to participants and via communication channels reserved for kite buggy sportsmen and sportswomen. The retrospective nature of the study deserves some reflection. First, assessing only serious and moderate injuries limits recall bias (the impact of which is greater with minor injuries).

Moreover, the fact that the sport depends on environmental variables means that many participants keep a journal of their sessions so they can better assess their performance and the suitability of different equipment in different wind conditions: this may have had a positive influence as regards injury dynamics data. Participants, in fact, provided very detailed information in this regard. The nature of extreme sports in general, often practiced alone or in small groups in remote places, adds challenges to the collection of data. This is a common problem when studying adventure/extreme sports [8], hence the retrospective nature of many such studies [5, 7, 10, 11, 15, 17, 19, 21, 23, 31, 35]. This study involved only active kiters; data for kiters who have decided not to practice the sport following an incident is unavailable. However, it should be acknowledged that athletes who do adventure/extreme sports usually return to active practice even after life-threatening or disabling injuries [10, 13, 19].

## Conclusions

Sport-specific knowledge is vital for the development of effective preparation, rehabilitation and safety systems. This study presented findings from a study on injury in kite buggying from a medical viewpoint. Injury-causing incidents in the sport stem from many variables typical of extreme sports, indeed, equipment and environmental variables contrast with the controlled circumstances of traditional sporting events [13]. The main dynamics in injury are liked to the OBE, and the shoulder is most likely to be injured. With regard to equipment, prevention needs to focus on the development and adoption of automatic release systems to prevent the OBE and protective clothing designed to protect the shoulder.

Participants recommend the introduction of formal training that includes an understanding of the relative meteorology and the establishment of designated kite buggying sites. Overall, while further studies are needed to add to the data produced by this survey, incident prevention in kite buggying is clearly a complex matter that requires a multi-sided approach that takes into account the relationship between the athlete, the activity and the environment.

## Abbreviations

AEKB: Asociación Española de Kite Buggy
AIS: Abbreviated Injury Scale
BCH: Buggy Club Holland
CIKB: Italian Kite Buggying Championship
FFCV: Fédération Francaise de Char à Voile
GPA: German Parakart Association Kitesailing
OBE: Out-of-buggy experience
PKA: Parakart Association
SKA: Swiss Kite Association
SPKA: Scottish Power Kite Association
